# Human Coronavirus 229E Uses ORF4/4a to Antagonize the Host Restriction Factor SERINC5

**DOI:** 10.1002/mco2.70785

**Published:** 2026-05-29

**Authors:** Qinya Xie, Sabrina Noettger, Jan Lawrenz, Sophie Stopper, Susanne Klute, Jan Münch, Dorota Kmiec, Qingxing Wang, Konstantin M. J. Sparrer, Frank Kirchhoff

**Affiliations:** ^1^ Institute of Molecular Virology Ulm University Medical Center Ulm Germany; ^2^ German Center for Neurodegenerative Diseases (DZNE) Ulm Germany

**Keywords:** endemic coronaviruses, innate immunity, SERINC, 229E ORF4a, 229E ORF4

## Abstract

Serine incorporator 5 (SERINC5) restricts the infectivity of various enveloped viruses, including HIV‐1 and severe acute respiratory syndrome coronavirus 2. However, these pandemic viral pathogens have evolved mechanisms to counteract this restriction. Here, we examined the impact of all five human SERINC family members on the seasonal human coronaviruses (hCoVs) 229E and OC43, which account for up to 15% of global mild respiratory infections and can cause severe disease in vulnerable individuals. Our data show that both exogenous and endogenous SERINC1, SERINC3, and SERINC5 significantly reduce OC43 infectivity in human lung and liver cells but have little, if any, effect on 229E. Functional analyses revealed that both the 130‐amino‐acid ORF4a protein encoded by most laboratory 229E strains and the full‐length 219 amino acid ORF4 protein encoded by clinical 229E isolates antagonize SERINC5 by promoting its relocalization to lysosomes and subsequent degradation. Finally, we show that endogenous SERINC5 expression in primary human lung cells inhibits infection by ORF4‐deficient but not wild‐type hCoV‐229E. In conclusion, several SERINC proteins restrict hCoV‐OC43, whereas hCoV‐229E efficiently counteracts SERINC‐mediated restriction by its ORF4/4a accessory proteins to ensure efficient production of fully infectious viral particles.

## Graphical abstract

The seasonal human coronaviruses 229E and OC43 are common causes of mild respiratory infections but can cause severe disease in vulnerable individuals. Serine incorporator 5 (SERINC5) efficiently inhibits OC43, while hCoV‐229E uses its ORF4/4a accessory proteins to efficiently counteract this restriction by promoting relocalization of SERINC5 to lysosomes and its subsequent degradation.

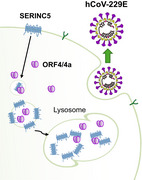

## 1 Introduction

Coronaviruses are a diverse family of enveloped RNA viruses that infect a broad range of vertebrate species, including humans [1, 2]. They recently gained enormous attention because the severe acute respiratory syndrome coronavirus 2 (SARS‐CoV‐2) caused the COVID‐19 pandemic [3]. Two other recently emerged human coronaviruses (hCoVs), SARS‐CoV‐1 and MERS‐CoV, were responsible for epidemic outbreaks with fatality rates of approximately 10 and 40%, respectively [4, 5]. While highly pathogenic coronaviruses have been extensively studied, much less is known about the endemic human common cold coronaviruses (ccCoVs), 229E, NL63, OC43, and HKU1. These pathogens are well adapted to their human host and are responsible for up to 30% of all mild to moderate upper respiratory tract infections worldwide [6, 7]. Although typically associated with mild symptoms, ccCoVs pose a substantial burden and costs to public health systems [8, 9]. In addition, they can cause severe complications in vulnerable populations, such as infants, elderly, and/or immunocompromised individuals [6]. A better understanding of the host factors that influence coronavirus infection and spread might help to develop new antiviral strategies and improve preparedness against future viral outbreaks.

Host restriction factors play key roles in viral zoonoses and subsequent spread in human populations [10, 11, 12, 13]. These structurally and functionally highly diverse cellular proteins are part of the intrinsic immune response and have the potential to inhibit viral pathogens at essentially all steps of their replication cycle [14, 15, 16, 17]. Among the numerous restriction factors identified to date, SERINC (serine incorporator) proteins, particularly SERINC3 and SERINC5, have emerged as inhibitors of a broad range of enveloped viruses, including HIV‐1 and SARS‐CoV‐2 [18, 19, 20]. It has been reported that incorporation of SERINC into the membrane of budding virions alters their lipid composition and envelope protein conformation, reducing their ability to fuse with the membrane of the host cell [21, 22, 23, 24, 25, 26]. Thus, SERINCs impair viral entry and limit the spread of enveloped viral pathogens [27].

While the antiviral function of SERINCs and viral countermeasures are well established in the context of HIV‐1 [18, 20], their role in restricting coronaviruses is less understood. SERINC5 has been reported to inhibit SARS‐CoV‐2 entry by blocking virus–cell fusion [19]. However, this effect is counteracted by the viral accessory protein ORF7a, which reduces SERINC5 incorporation into budding virions [19]. In addition, it has been suggested that SARS‐CoV‐2‐encoded small RNAs suppress SERINC5 expression [28]. Whether ccCoVs are inhibited by SERINCs, or have evolved effective countermeasures, is currently unknown. Studies on the impact of SERINC proteins on ccCoVs may not only advance our understanding of what made these viruses so successful, but also provide insights into host–virus interactions and viral evolution.

To date, coronaviruses have been transmitted from animals to humans on at least seven occasions and ccCoVs share many structural and functional similarities with highly pathogenic MERS and SARS coronaviruses [29, 30, 31]. Thus, insights into how SERINCs restrict ccCoVs and how these viruses evade or counteract this restriction may have implications for other current as well as newly emerging coronaviruses. Here, we examined the Alphacoronavirus 229E and the Betacoronavirus OC43, which both originated from zoonoses but differ in their genus, reservoir and intermediate hosts, timing of transmission, receptor usage, and accessory‐gene features [6, 32, 33, 34, 35]. We show that several SERINC proteins restrict OC43. In contrast, 229E is resistant because its ORF4/4a proteins efficiently antagonize human SERINC proteins.

## 2 Results

### 2.1 Overexpression of SERINC1, 3, and 5 Impairs Infectivity of OC43 but Not 229E

**FIGURE 1:**
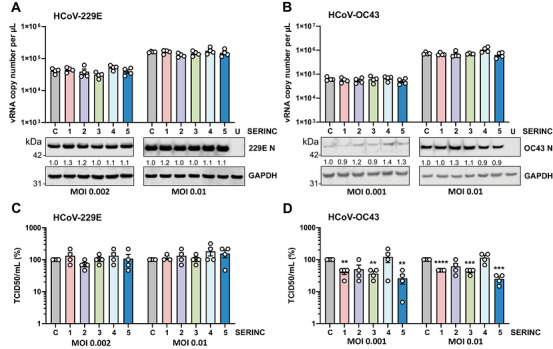
Overexpression of SERINC1, 3, and 5 impairs infectivity of hCoV‐OC43 but not hCoV‐229E. (A and B) Huh7 cells transiently transfected with empty or SERINC encoding pBJ6 vectors were infected with hCoV‐229E (A) or hCoV‐OC43 (B) at the indicated MOIs. At 30 hpi (A) or 48 hpi (B), supernatants and cell lysates were collected for qRT‐PCR and western blot analysis, respectively. (C) Supernatants of Huh7 cells treated as described in (A) were harvested for TCID_50_ determination at 30 h postinfection. (D) Huh7 cells were treated as described in (B) and supernatants harvested for TCID_50_ assay at 72 h postinfection. Shown are mean values ± SEM from three or four independent experiments. Statistical significance compared with the mock control was assessed using unpaired *t*‐test with Welch's correction. **p ≤ 0.01, ***p ≤ 0.001, ****p ≤ 0.0001.

To assess the sensitivity of 229E and OC43 to SERINC proteins, we infected Huh7 cells transiently transfected with constructs encoding the five different members of the human SERINC family [36] or an empty control vector at multiplicities of infection (MOIs) of 0.01 and 0.002, respectively. To minimize overexpression artifacts, we used the low‐expression pBJ6 vector, which produces ∼100‐fold less SERINC protein than conventional CMV‐driven plasmids [18]. With the exception of SERINC4, which is known to be unstable [37], all SERINC proteins were expressed at detectable albeit varying levels (Figure S1A). None of the SERINC proteins had significant effects on the intracellular expression levels of the 229E and OC43 Nucleocapsid (N) proteins or on viral RNA copy numbers in the culture supernatants (Figure 1A,B). Overexpression of SERINC proteins did not affect the infectivity of 229E (Figures 1C and S1B). In contrast, SERINC1, 3, and 5 reduced the infectious titers of OC43 by 50–80% (Figure 1D). This reduction remained significant when the infectivity was normalized to the viral RNA copy numbers (Figure S1C). Thus, SERINCs did not impact the production of hCoV‐OC43 particles but reduced their infectiousness, while hCoV‐229E was resistant.

### 2.2 Endogenous SERINC1, 3, 4, and 5 Restrict OC43 in Human Lung and Liver Cells

To determine whether endogenous SERINC proteins affect the infectivity of 229E or OC43, we first analyzed SERINC mRNA expression in Huh7, A549, Caco2, and Calu3 cells as well as in primary normal human lung fibroblasts (NHLFs) (Figure S2A). Huh7 and Caco2 are liver and intestinal epithelial cell lines, respectively, and frequently used in coronavirus research [38]. A549 and Calu‐3 are derived from a human lung adenocarcinoma and the bronchial epithelium [39, 40]. Finally, NHLF represent normal primary lung fibroblasts. All five cell types expressed high levels of SERINC1 and 3 mRNAs, while SERINC2 and 4 transcripts were only detected at low levels (Figure S2A). mRNA encoding for SERINC5, representing the best characterized antiviral factor, was usually detected at intermediate levels. Consistent with previous findings [18, 20], neither viral infection nor IFN‐β treatment altered SERINC3 and SERINC5 expression, while IFN‐β induced a ∼20‐fold increase in expression of 2′‐5′‐oligoadenylate synthetase 1, a well‐known IFN‐stimulated gene used as positive control (Figure S2B). Expression of SERINC4 was moderately increased by both hCoV infection and IFN‐β, but the significance of this effect is difficult to assess because SERINC4 mRNA levels were generally very low.

**FIGURE 2:**
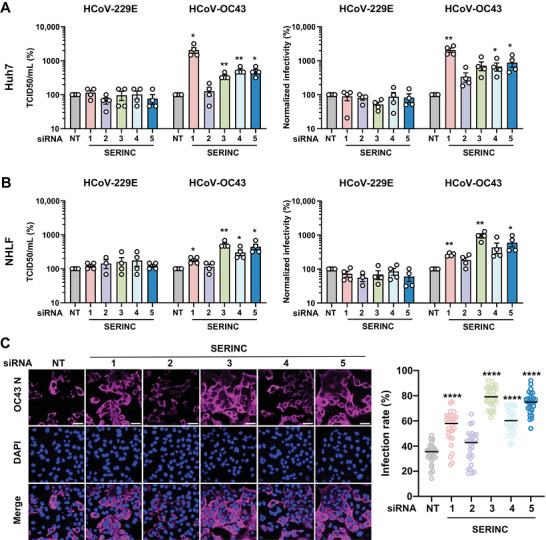
Endogenous SERINC1, 3, 4, and 5 restrict infectivity of hCoV‐OC43 but not hCoV‐229E. (A) Huh7 cells transfected with nontargeting or SERINC‐specific siRNA were infected with hCoV‐229E at an MOI of 0.001 or hCoV‐OC43 at MOI 0.01. Supernatants were harvested at 30 or 72 hpi for qRT‐PCR and TCID_50_. Normalized infectivity was calculated as TCID_50_/vRNA copy number, with the vector control set to 100%. (B) NHLF cells transfected with nontargeting or SERINC‐specific siRNAs were infected with hCoV‐229E (MOI 0.02)) or hCoV‐OC43 (MOI 0.002). Supernatants were collected at 36 hpi (hCoV‐229E) or 72 hpi (hCoV‐OC43) for qRT‐PCR and TCID_50_. Normalized infectivity was calculated as TCID_50_/vRNA copy number, with the vector control set to 100%. (C) Supernatants from (B) were used to infect A549 cells. Viral infection was assessed by immunofluorescent staining of the hCoV‐OC43 nucleocapsid (purple) and DAPI‐stained nuclei (blue). Scale bar: 40 µm. Infection rates were quantified using image J. A total of 28 images (seven images from each independent experiment) per condition were included for analysis. Shown are mean values ± SEM from three or four independent experiments. Statistical comparisons with mock control were performed using unpaired *t*‐test with Welch's correction. *p ≤ 0.05; **p ≤ 0.01; ****p ≤ 0.0001.

To explore functional effects, we used siRNAs to deplete individual SERINCs in Huh7 and A549 cells, followed by infection with 229E or OC43. Knock‐down (KD) efficiencies were >80% and specific for each target (Figure S2C–F). In agreement with the overexpression data (Figure 1), SERINC depletion had no effect on 229E infectivity in Huh7 cells (Figure 2A). In contrast, silencing SERINC1 increased OC43 titers ∼20‐fold, while KD of SERINC3, 4, and 5 led to ∼7‐fold increases (Figure 2A). Again, this effect was due to enhanced virion infectivity and not production. Similar effects were observed in A549 cells: siRNAs targeting SERINC1, 3, and 5 increased OC43 infectivity up to 10‐fold, while silencing of the poorly expressed SERINC2 and 4 mRNAs had lesser effects (Figure S3).

To validate these findings in primary cells, we silenced the five SERINCs in NHLFs (Figure S2E). In line with the cell line data, SERINC silencing had no significant effect on the infectivity of 229E (Figure 2B). In contrast, depletion of SERINC3 and 5 increased the infectivity of OC43 in NHLF cells by ∼9‐ and 6‐fold, respectively (Figure 2B). Passaging of the virus produced on NHLF cells to A549 cells confirmed that KD of SERINCs 1, 3, 4, and 5 increased viral Nucleocapsid (N) protein detection in A549 cells indicating increased infectious OC43 yields (Figure 2C). Altogether, these results showed that endogenous SERINCs restrict OC43 in human lung and liver cells, while hCoV‐229E is fully resistant.

### 2.3 Evolutionary Conservation and Determinants of SERINC‐Mediated OC43 Restriction

**FIGURE 3:**
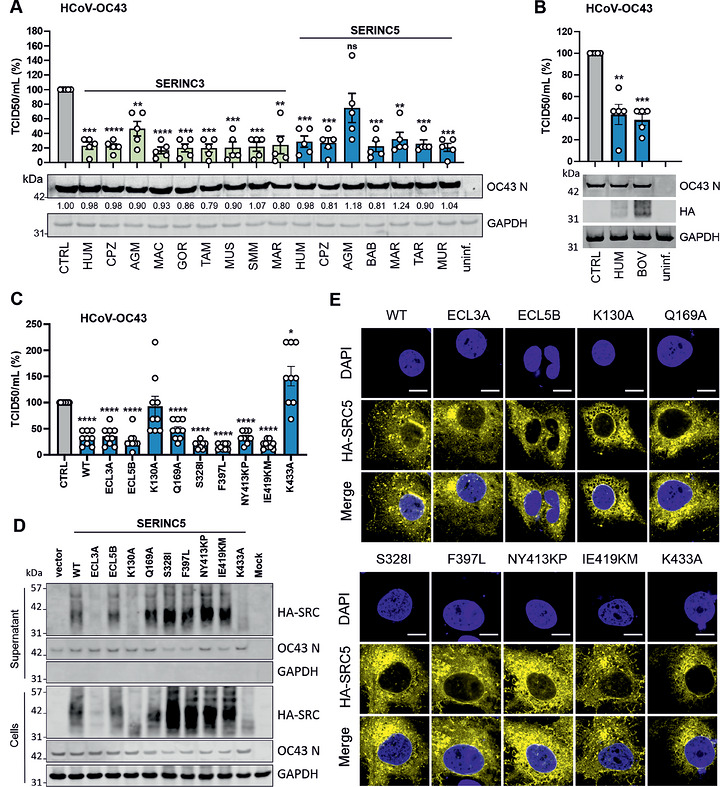
Conservation and determinants of SERINC‐mediated restriction of hCoV‐OC43. (A) Huh7 cells transiently transfected with SERINC3 or SERINC5 orthologs from various species were infected with hCoV‐OC43 (MOI 0.01). Supernatants and cell lysates were harvested for TCID_50_ and western blot analysis at 72 hpi. Species abbreviations: humans (HUM), chimpanzees (CPZ), African green monkeys (AGM), rhesus macaques (MAC), gorillas (GOR), tamarins (TAM), mustached monkeys (MUS), sooty mangabeys (SMM), common marmosets (MAR), olive baboons (BAB), and mice (MUR). (B) Huh7 cells were transfected with constructs expressing human or bovine (BOV) SERINC5, infected with hCoV‐OC43, and virus production determined as described in panel A. (C and D) Huh7 cells transiently transfected with wild‐type or mutant SERINC5 constructs were infected with hCoV‐OC43. Supernatants and whole cell lysates were collected at 72 hpi for TCID_50_ (C) and western blot analyses of cell lysates and purified virions (D). Panels A–C show mean values ± SEM from five or nine independent experiments. Statistical significance of differences compared with the mock control was assessed using unpaired *t*‐test with Welch's correction. *p ≤ 0.05, **p ≤ 0.01. ***p ≤ 0.001, ****p ≤ 0.0001. (E) Huh7 cells transiently transfected with wild‐type or mutant SERINC5 were fixed for immunofluorescence staining. Nuclei were stained with DAPI (blue), wild‐type or mutant SERINC5 were stained with an HA antibody (yellow). Scale bar: 10 µm.

SERINCs are highly conserved in sequence and antilentiviral function, with no signs of positive selection [41, 42, 43]. To determine whether the activity of SERINCs against OC43 is evolutionarily conserved, we tested 16 SERINC3 and SERINC5 orthologs from humans, non‐human primates, and mice. Most SERINC3/5 orthologs reduced OC43 titers in Huh7 supernatants by ∼80%, without affecting viral N protein expression levels in infected cells (Figure 3A). This inhibitory effect was observed for all primate‐derived and mouse SERINC proteins. The exceptions were SERINC3 and SERINC5 from African green monkeys (AGM), which only reduced infectious OC43 yields by ∼50 and 25%, respectively. It has been suggested that OC43 originated in rodents and was transmitted into humans via cattle as intermediate hosts [6, 44]. Thus, we generated a construct expressing bovine SERINC5 and found that it is as active as the human orthologue in restricting OC43 (Figure 3B). Altogether, these results show that SERINC‐mediated restriction of OC43 is conserved from mice to men.

To identify molecular determinants of SERINC‐mediated restriction of OC43, we tested nine previously described SERINC5 mutants [45]. The mutations were designed based on the structure and amino acid sequence conservation between SERINC5 and SERINC2, which lacks antiviral activity. Mutation of K130A and K433A, known to disrupt plasma membrane localization [45], impaired antiviral activity (Figure 3C). These mutations also reduced SERINC5 protein levels in both cell lysates and supernatants (Figure 3D). In contrast, the ECL3A mutant, which contains the ECL3 of SERINC2, retained full anti‐OC43 activity (Figure 3C). This chimeric SERINC5 protein also showed reduced expression (Figure 3D). Thus, reduced protein levels alone do not account for loss of function. Most SERINC proteins were detected in the juxtanuclear and perinuclear regions, as well as in punctate structures throughout the cytoplasm (Figure 3E). This agrees with data showing that SERINC5 localizes to the endoplasmic reticulum‐Golgi intermediate compartment (ERGIC) [19], the site of budding and subsequent release by exocytosis for OC43 and other coronaviruses [46, 47]. This punctate pattern, which is characteristic for the ERGIC, was altered for the K130A and K433A mutant forms of SERINC5 (Figure 3E). Consistent with their reduced expression, these two forms appeared to localize to lysosomes, although further studies are required for definitive proof. In contrast to the reported impact on HIV‐1 [45], exchanges in ECL3 and ECL5, as well as mutations of Q169A, S328I, F397L, NY413KP, and IE419KM, did not impair OC43 restriction. Altogether, these results indicate that an ERGIC‐like localization pattern of SERINC5 is associated with efficient restriction of OC43.

### 2.4 229E and OC43 Infection Reduce SERINC Expression

**FIGURE 4:**
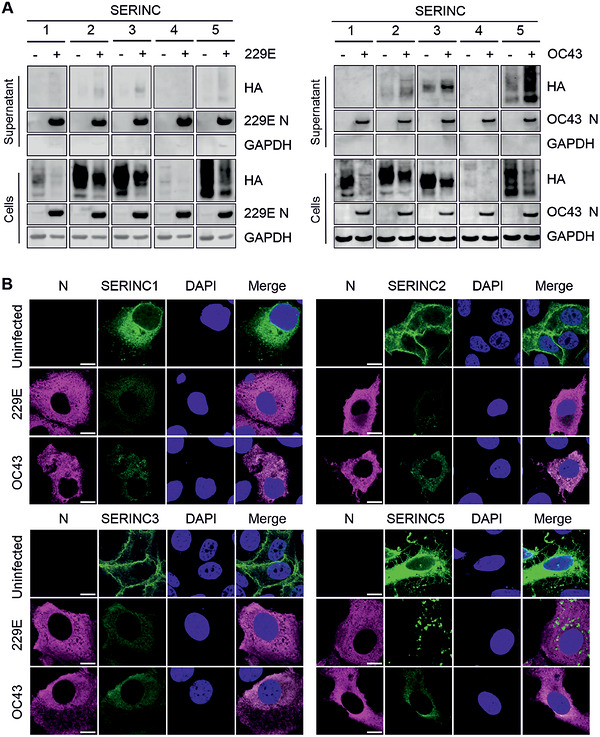
hCoV‐229E and hCoV‐OC43 reduce exogenous SERINC expression levels. (A) Huh7 cells transiently transfected with HA‐tagged SERINCs were infected with hCoV‐229E (MOI 0.001) or hCoV‐OC43 (MOI 0.01). Whole cell lysates and supernatants were harvested at 3 days postinfection (dpi) for western blot. Shown are the western blot analyses of cell lysates and purified virions representative for four independent experiments. (B) Huh7 cells transfected with GFP‐tagged SERINC1, SERINC2, SERINC3, and SERINC5 were infected with hCoV‐229E (MOI 0.01), hCoV‐OC43 (MOI 0.1), or left untreated. Immunofluorescence staining was performed at 2 dpi. Nucleocapsid proteins (purple) of hCoV‐229E or hCoV‐OC43 were stained to indicate infection; nuclei were stained with DAPI (blue). Scale bar: 20 µm. Results are representative of at least three independent experiments.

To investigate whether viral infection affects SERINC expression and subcellular localization, we transfected Huh7 cells with constructs encoding GFP‐tagged SERINC proteins and subsequently infected them with 229E or OC43. As previously shown (Figure S1A), SERINC2, 3, and 5 were expressed at higher levels than SERINC4, while SERINC1 showed an intermediate phenotype (Figure 4A). We found that all SERINC proteins were markedly reduced in extracts derived from infected cell cultures compared with uninfected controls. In contrast, viral infection increased the levels of SERINC2, 3, and 5 in the supernatants, especially those obtained from OC43‐infected cultures, suggesting potential incorporation into viral particles (Figure 4A). Confocal microscopy analyses confirmed reduced expression of GFP‐SERINC1, 2, 3, and 5 fusions in cells infected with 229E and OC43 (Figure 4B). We did not analyze SERINC4 because it was generally poorly expressed. Both 229E and OC43 reduced the overall expression levels of GFP‐SERINC proteins but this effect and relocalization from the cell surface to intracellular compartment was stronger in 229E‐infected cells. While OC43 removed SERINCs (especially 2 and 3) from the plasma membrane, only 229E induced accumulation of SERINC5 in large intracellular vesicles (Figure 4B), suggesting a virus‐specific mechanism of subcellular redistribution.

### 2.5 229E Induces Lysosomal Degradation of SERINC5

**FIGURE 5:**
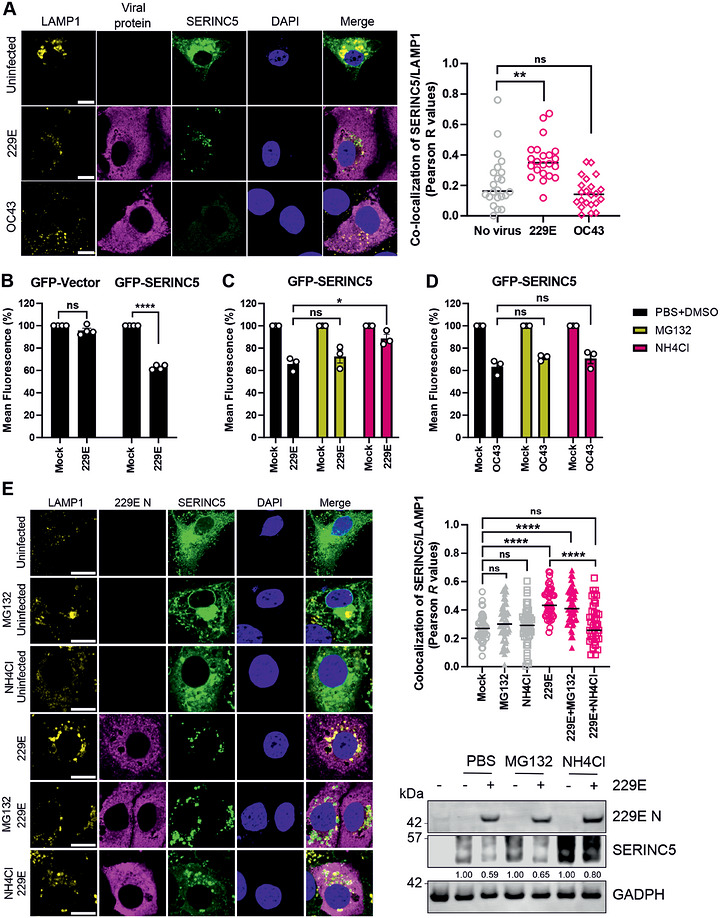
SERINC5 degradation by hCoV‐229E infection is lysosome dependent. (A) Huh7 cells transiently transfected with GFP‐SERINC5 were infected with hCoV‐229E (MOI 0.01), hCoV‐OC43 (MOI 0.1), or left untreated. Immunofluorescence staining was performed at 2 dpi. Viral infection was detected via nucleocapsid staining (purple), and lysosomes were marked with LAMP1 (yellow). Colocalization of GFP‐SERINC5 with LAMP1 was quantified using Huygens software (*n* = 22 cells). Scale bar: 20 µm. (B) Huh7 cells transiently transfected with vectors expressing GFP alone or GFP‐SERINC5 were infected with hCoV‐229E or left untreated. Mean GFP fluorescence intensity in live cells was measured by flow cytometry at 2 dpi. (C and D) Huh7 cells transfected with GFP‐SERINC5 were infected with hCoV‐229E (C), hCoV‐OC43 (D), or left uninfected, followed by treatment with proteasome inhibitor MG132 (20 µM, 6 h) or lysosome inhibitor NH_4_Cl (10 mM, 18 h). Cells were harvested at 48 h postinfection for flow cytometry analysis. (E) Huh7 cells transiently transfected GFP‐SERINC5 were infected with hCoV‐229E, followed by MG132 treatment for 6 h or NH_4_Cl treatment for 18 h. At 48 hpi, cells were fixed for immunofluorescence staining or harvested for western blot. Scale bar: 20 µm. A total of 45 cells were analyzed for colocalization of GFP‐SERINC5 and LAMP1 using Huygens. Shown are mean values ± SEM from three or four independent experiments. Statistical significance compared with the mock control was assessed using unpaired *t*‐test with Welch's correction. *p ≤ 0.05, **p ≤ 0.01. ***p ≤ 0.001, ****p ≤ 0.0001.

We next investigated whether 229E or OC43 counteract SERINC5 by promoting its lysosomal or proteasomal degradation. SERINC5 accumulated in LAMP‐1‐positive vesicles and showed strong colocalization with this lysosomal marker in the presence of 229E but not OC43 infection (Figure 5A). Control experiments confirmed that 229E leads to downregulation of SERINC5‐GFP, but not GFP alone (Figure 5B). Flow cytometry results showed that degradation of SERINC5‐GFP in 229E‐infected cells was not affected by the proteasome inhibitor MG132 but significantly inhibited by NH_4_Cl, which inhibits the autophagy/lysosomal pathway (Figure 5C). In contrast, OC43‐mediated reduction of GFP‐SERINC5 was not affected by either inhibitor (Figure 5D). Confocal microscopy analyses confirmed that NH_4_Cl prevented 229E‐mediated recruitment of SERINC5 to LAMP‐1 and lysosomal degradation (Figure 5E). These results suggest that 229E induces lysosomal degradation of SERINC5.

### 2.6 229E ORF4a Mediates SERINC5 Relocalization and Degradation

**FIGURE 6:**
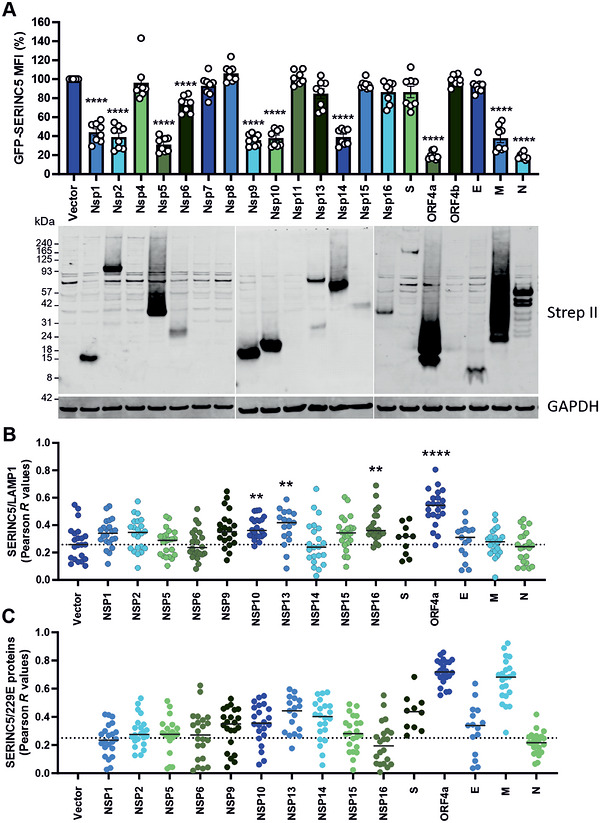
hCoV‐229E ORF4a mediates SERINC5 relocalization and degradation. (A) Huh7 cells were cotransfected with constructs expressing GFP‐SERINC5 and the indicated hCoV‐229E proteins. At 2 dpi, cells were analyzed by flow cytometry staining or western blot. Shown are mean GFP fluorescence intensities in live cells, normalized to vector control. Data represent two biological replicates from four independent experiments. (B and C) Colocalization analysis of GFP‐SERINC5 with LAMP1 or with Strep II‐tagged viral proteins in Huh7 cells. A total of 10 to 22 cells in each group were included for analysis. Statistical significance compared with the mock control was determined using unpaired *t*‐test with Welch's correction. **p ≤ 0.01, ****p ≤ 0.0001.

To identify which 229E protein counteracts SERINC5, we cotransfected Huh7 cells with a vector expressing GFP‐SERINC5 and constructs encoding 20 different StrepII‐tagged 229E proteins. Western blot confirmed expression of most viral proteins (Figure 6A). Unexpectedly, flow cytometric analyses showed that nine of the 20 229E proteins clearly reduced expression levels of the GFP‐SERINC5 fusion, with ORF4a and N exerting the strongest effects (Figure 6A). Confocal microscopy confirmed that ORF4a, M and N markedly reduced the levels of GFP‐SERINC5 expression in Huh7 cells (Figure S4). For Nsp1, this was expected since it is known to suppress cellular protein expression by inhibiting ribosomal translation [48, 49]. Further analyses showed that Nsp10, Nsp13, Nsp16, and ORF4a increased colocalization of SERINC5 with the LAMP1 lysosomal marker (Figure 6B). Since ORF4a had the strongest effect and also colocalized with SERINC5 (Figure 6C), it was selected for in‐depth follow‐up.

**FIGURE 7:**
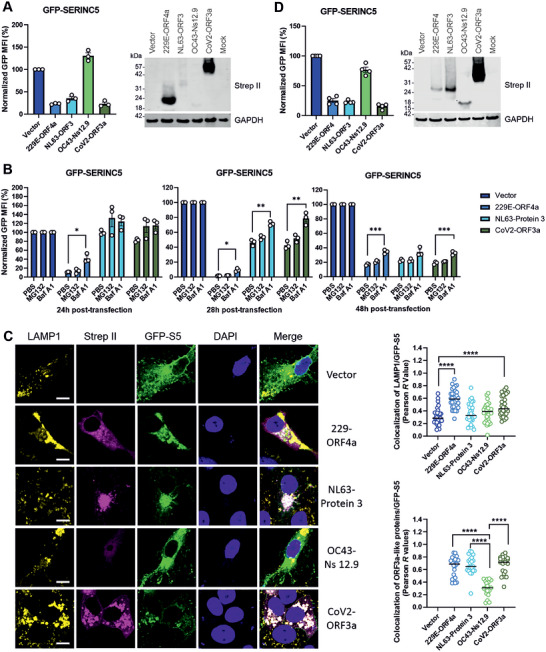
229E‐ORF4, NL63‐ORF3, and CoV‐2‐ORF3a induce SERINC5 degradation. (A) Huh7 cells were cotransfected with constructs expressing GFP‐SERINC5 and the indicated ORF3a‐like proteins. At 2 dpi, cells were harvested for flow cytometry staining or western blot. Data show the mean fluorescence intensity (MFI) in Strep II positive cells, compared with the vector control. (B) Huh7 cells were cotransfected as in (A), then treated with Baf A1 (200 nM, 12 h), MG132, 20 µM, 4 h), or left untreated. Flow cytometry was performed at 24 h, 28 h, or 48 h post‐transfection. Data represent the MFI of Strep II‐positive cells, normalized to the vector control. (C) Huh7 cells cotransfected with constructs expressing GFP‐SERINC5 and the indicated ORF3a‐like proteins were fixed for immunofluorescence staining at 2 dpi. Strep II (purple) marks ORF3a‐like protein expression; LAMP1 (yellow) labels lysosomes; DAPI (blue) stains nuclei. Scale bar: 20 µm. Colocalization of GFP‐SERINC5 with LAMP1 was analyzed using Huygens in 30 cells per condition; colocalization with ORF3a‐like proteins was assessed in 21 cells per condition. (D) Huh7 cells were cotransfected with constructs expressing GFP‐SERINC5, full‐length 229E‐ORF4 or the indicated ORF3a‐like proteins as in (A), except that a different vector was used (pTwist instead of pLVX‐EF1 alpha). At 2 dpi, cells were harvested for flow cytometry staining or western blot. The data shown represent the MFI in strep II positive cells, normalized to the vector control. Statistical significance compared with the mock control was determined using unpaired *t*‐test with Welch's correction. *p ≤ 0.05, **p ≤ 0.01. ***p ≤ 0.001, ****p ≤ 0.0001.

229E‐ORF4a protein is functionally analogous to the SARS‐CoV‐2 ORF3a protein, and both act as viroporins [50, 51]. The corresponding accessory proteins encoded between the spike and envelope genes in NL63 and OC43 are NL63‐ORF3 and OC43‐Ns12.9, respectively (Figure S5A). To further assess the efficiency of 229E‐ORF4a‐mediated SERINC5 antagonism, we compared its effect with those of the NL63‐ORF3, OC43‐Ns12.9, and SARS‐CoV‐2 ORF3a. The remaining proteins were chosen because they are expressed from a similar genomic location or show homology to 229E ORF4a [50, 51, 52, 53]. 229E‐ORF4a, NL63‐ORF3, and CoV2‐ORF3a but not OC43‐Ns12.9 efficiently reduced GFP‐SERINC5 expression levels (Figure 7A).

229E‐ORF4a already efficiently reduced expression of GFP‐SERINC5 1 day posttransfection, while NL63 protein 3 and CoV2‐ORF3a required 2 days to achieve similar efficiencies (Figure 7B). In further support of lysosomal degradation, inhibition of lysosomes using Bafilomycin A1 but not the proteasome inhibitor MG132 reduced the inhibitory activities. In agreement with this, 229E‐ORF4a and to a lesser extent SARS‐CoV2‐ORF3a relocated GFP‐SERINC5 to lysosomal compartments (Figure 7C). In contrast, the NL63‐ORF3 did not induce relocalization although it also colocalized with SERINC5 (Figure 7C). Notably, all viral accessory proteins that downmodulated GFP‐SERINC5 expression (229E‐ORF4, NL63‐ORF3, and CoV‐2‐ORF3a) increased the number of autophagosomes in flow‐cytometry‐based assay systems (Figures 7D and S6B) [54], indicating activation of the lysosomal degradation pathway. Altogether, these data show that hCoVs developed several antagonists of human SERINC proteins and indicate that the 229‐ORF4a protein is particularly effective against SERINC5.

The ORF4a gene present in laboratory 229E strains contains a 2‐nucleotide deletion compared with most clinical isolates [55]. This deletion introduces an early stop codon and truncates the ORF4 protein from 219 to 130 amino acids (Figure S6A). Importantly, the full‐length 229E‐ORF4 protein reduced expression of GFP‐SERINC5 fusions as efficiently as ORF4a (Figure 7D). In addition, ORF4 also relocalized SERINC5 to lysosomes (Figure S6C). Altogether, these results show that as previously reported for the viroporin function [50], the ability to target SERINC5 for lysosomal degradation is conserved between ORF4 and ORF4a.

### 2.7 ORF4 is Critical for SERINC5 Resistance of 229E

**FIGURE 8:**
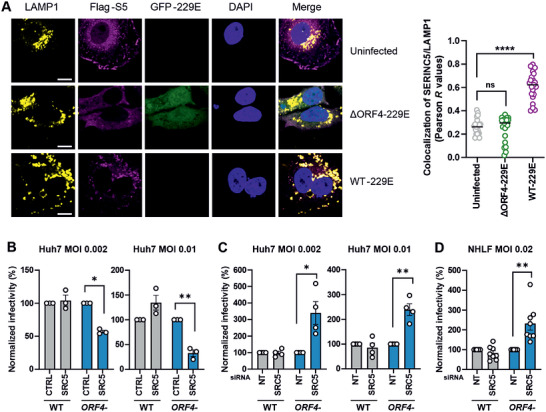
hCoV‐229E ORF4 is critical for SERINC5 resistance of hCoV‐229E. (A) Huh7 cells were transfected with a construct expressing SERINC5‐iFLAG followed by infection with wild‐type or ORF4‐deleted GFP‐hCoV‐229E. At 2 dpi, cells were fixed for immunofluorescence staining. SERINC5‐iFLAG was detected via anti‐FLAG staining (purple); LAMP1 (yellow) marks lysosomes; DAPI (blue) stains nuclei. Scale bar: 20 µm. Colocalization of GFP‐SERINC5 with LAMP1 was quantified using Huygens (*n* = 21 cells per condition). (B) Huh7 cells transiently transfected with empty or SERINC5 encoding vector were infected with wild‐type or GFP hCoV‐229E at indicated MOIs. At 36 hpi, supernatants and cell lysates were harvested for qRT‐PCR, TCID_50_, and western blot. Normalized infectivity was calculated as TCID_50_/vRNA copy number, with the vector control set to 100%. (C) Huh7 cells transfected with nontargeting or siRNA or SERINC5‐targeting siRNAs were infected with wild‐type or GFP hCoV‐229E at the indicated MOIs. Supernatants were harvested for qRT‐PCR and TCID_50_ at 36 hpi. (D) NHLF transfected with nontargeting or SERINC5‐targeting siRNAs were infected with wild‐type or GFP hCoV‐229E (MOI 0.02). Supernatants were collected at 48 hpi for qRT‐PCR and TCID_50_. Normalized infectivity was calculated as TCID_50_/vRNA copy number, with the vector control set to 100%. Shown are mean values ± SEM from three to eight independent experiments. Statistical significance compared with the mock control was determined using unpaired *t*‐test with Welch's correction. *p ≤ 0.05, **p ≤ 0.01, ****p ≤ 0.0001.

To determine whether ORF4 is critical for resistance of genuine 229E to SERINC restriction, we took advantage of a reporter virus containing the *GFP* gene in place of *ORF4* [56, 57]. Infection of Huh7 cells revealed that lack of ORF4 impaired the ability of hCoV‐229E to relocate SERINC5 to lysosomal compartments (Figure 8A). Overexpression of SERINC5 in Huh7 cells reduced the infectiousness of ORF4a defective 229E by 50–70% but had no significant effect on the wild‐type virus (Figures 8B and S7A). Conversely, KD of endogenous SERINC5 expression in Huh7 cells (Figure S7B) significantly increased the infectivity of *ORF4*‐defective but not of wild‐type hCoV‐229E (Figure 8C). Importantly, KD of SERINC5 in primary NHLF also increased the infectiousness of 229E produced in the absence but not in the presence of ORF4 (Figure 8D). Thus, 229E uses its ORF4 protein to avoid SERINC5 restriction in primary human lung cells.

## 3 Discussion

Innate restriction factors play key roles in limiting viral spread and pathogenesis. Here, we show that several members of the human SERINC family, especially SERINC1, 3, and 5, reduce the infectiousness of the ß‐coronavirus hCoV‐OC43, while the α‐coronavirus hCoV‐229E is fully resistant. Both viruses, however, markedly reduce the levels of SERINC protein in infected cells. At least in part, this is most likely due to expression of NSP1, which is known to efficiently suppress host protein synthesis in SARS‐CoV‐2 and MERS‐CoV infections [48, 49, 58]. Unexpectedly, overexpression of several additional hCoV‐229E proteins (Nsp2, Nsp5, Nsp9, Nsp10, Nsp14, ORF4a, M, and N) also reduced SERINC expression in transfected Huh7 cells. However, only ORF4a efficiently induced lysosomal relocalization and degradation of SERINC5, the best characterized antiviral member of this family. Functional analyses confirmed that the ability to counteract SERINC5 is conserved between ORF4a and full‐length ORF4 and that this function is essential for hCoV‐229E resistance in human lung and liver cells.

SERINC5 has been shown to inhibit SARS‐CoV‐2 entry by blocking virus–cell fusion, although this effect is partially antagonized by the viral protein ORF7a which is unique to SARS‐CoV‐1 and 2 [19]. We found that SERINC proteins also reduce the infectivity of OC43, which also belongs to the ß‐coronavirus subfamily. Inhibition was confirmed in both overexpression and KD experiments in various human cell lines and primary human lung fibroblasts. Although only OC43 was restricted, the levels of SERINC proteins were reduced in cells infected with both the α‐coronavirus 229E, as well as OC43. This agrees with the previous data showing that NSP1 proteins of various coronaviruses inhibit translation of host proteins [46, 48, 49].

While both 229E and OC43 reduce cellular SERINC expression, only 229E targets SERINC5 for lysosomal degradation. This mechanism is reminiscent of HIV‐1 Nef‐mediated downmodulation of SERINC5 via the autophagy–lysosomal pathway [59]. Our data add to the accumulating evidence suggest that coronaviruses have evolved diverse mechanisms to antagonize SERINCs [19, 28]. This is reminiscent to retroviruses, which can avoid restriction by inducing lysosomal or proteasomal degradation of SERINC5 [59, 60], as well as changes in the HIV‐1 envelope protein [61, 62]. The fact that different viruses have evolved various countermeasures highlights the important role of SERINC restriction. It has been reported that the potency of Nef‐mediated SERINC5 antagonism correlates with the prevalence of simian immunodeficiency viruses in the wild [42]. In contrast, OC43 is more prevalent than 229E but the latter is associated with higher pathogenicity, especially in elderly and immunocompromised individuals [63, 64, 65]. OC43 likely originated from rodents and is thought to have been transmitted from cattle as intermediate hosts to humans around the 1890s, while 229E crossed into humans from camels in the mid‐20th century [6, 66, 67]. The earlier zoonotic transmission of OC43 helps to explain its higher prevalence. How OC43 downmodulates some SERINCs from the cell surface and whether sensitivity to SERINC‐mediated restriction influences viral transmission or disease severity warrants further study.

Unlike other antiviral restriction factors, SERINC3 and 5 are evolutionary conserved and do not show evidence for positive selection driven by an evolutionary arms race with viral pathogens [43]. As previously observed for primate lentiviruses [41, 42], it is thus conceivable that the ability of SERINC3 and 5 to restrict OC43 is species‐independent and conserved between men and mice (Figure 3A). Similar to the effects on HIV‐1 [45], SERINC5 mutants that do not localize at the plasma membrane failed to inhibit hCoV‐OC43 (Figure 4). Thus, cellular surface expression and virion incorporation seem critical for restriction of both coronaviruses and retroviruses. In contrast, mutations that were restriction‐defective against HIV‐1 despite virion incorporation, including exchanges in ECL3 and ECL5, as well as mutations of NY413KP and IE419KM, were fully active against hCoV‐OC43. Thus, unlike the effect of SERINC5 on HIV‐1, restriction of OC43 and possibly other coronaviruses does not seem to depend on specific conformations of SERINC5. Altogether, these findings further support that antiviral activity is a conserved feature of SERINCs, and most likely plays a role in the host defense against coronaviruses across various mammalian species.

Previously studies largely focused on SERINC5 and to a lesser extent on SERINC3. Here, we performed broader analyses of all five human SERINC proteins. In agreement with previous data [37], SERINC4 mRNA and protein were both expressed at low levels compromising meaningful analyses. Unexpectedly, however, KD of endogenous SERINC4 expression increased OC43 infectivity in some cell types suggesting that it displays antiviral activity. More notably, SERINC1, that shows similar structural features and subcellular localization as SERINC5 [68], strongly inhibited OC43 in Huh7 cells. Similar to previous data on HIV‐1 [22, 45, 69], SERINC3 and 5 strongly inhibited OC43, while SERINC2 lacks antiviral activity. Currently, little is known about the expression, localization and function of SERINC1, 2, and 4. Thus, further studies on the contribution of different SERINC proteins to virus restriction seem warranted although SERINC5 clearly plays a key role.

In conclusion, our results demonstrate that several human SERINC proteins restrict hCoV‐OC43 but not hCoV‐229E. The reason for this is that the 229E‐ORF4/4a proteins but not the OC43‐Ns12.9 proteins present at the same genomic location induce lysosomal relocalization and degradation of SERINC5. Our results add to the evidence that SERINCs are important components of innate immunity although they don't share features of other known antiviral factors, such as inducibility by IFNs or evidence for positive selection. However, coronaviruses have evolved diverse mechanisms to evade this restriction, and understanding these in greater detail may provide insights into their pathogenicity and transmission fitness.

## 4 Materials and Methods

### 4.1 Plasmids and Viruses

Full‐length human SERINC1 and SERINC4 were cloned (from Sinobiological Cat No. HG14071‐CY and Cat No. HG28785‐UT) into pBJ6 expression construct via *Not*I/*Eco*RI sites. SERINC‐expressing constructs pBJ6‐SERINC2‐HA, pBJ6‐SERINC3‐HA, pBJ6‐SERINC5‐HA, SERINC2‐GFP, SERINC3‐GFP, SERINC5‐GF,P and pEGFP‐N1 construct were kindly provided by Massimo Pizzato. SERINC1‐GFP construct was purchased from Sinobiological (Cat No. HG14071‐ACG). SERINC3 and SERINC5 variants from primates and mice were generated as described before [42], full‐length bovine SERINC5 purchased from Genscript (XM_027552960.1) was cloned into the same vector via *Not*I/*Eco*RI sites. SERINC5‐iFLAG‐HA and different mutants were gifts from Peter Cherepanov [45]. hCoV‐229E protein encoding constructs in a Gateway‐compatible Entry vector [70] were kindly provided by Pascal Falter‐Braun and subsequently cloned into the pLVX backbone using Gibson assembly via *Not*I/*Eco*RI sites. Expression constructs for the empty vector, hCoV‐229E‐ORF4, hCoV‐NL63‐ORF3, hCoV‐OC43‐Ns12.9, and SARS‐CoV‐2‐ORF3a in pTWIST backbone were obtained from TWIST Bioscience. hCoV‐229E and hCoV‐OC43 were purchased from ATCC (Cat No. VR‐740TM and Cat No. CR‐1558TM) and propagated as described [71]. GFP‐hCoV‐229E which lacks ORF4 was a gift from Volker Thiel.

### 4.2 Cell Culture

All cells were maintained at 37°C in a humidified atmosphere with 5% CO2. Huh7, A549, Caco2, and NHLF cells were cultured in Dulbecco's modified Eagle medium (DMEM) supplemented with 100 µg/mL streptomycin, 100 units/mL penicillin, 2 mM L‐glutamine, 1 mM nonessential amino acids (NEAA), and 10% heat‐inactivated fetal calf serum (FCS) or 2% FCS for infection. Calu3 cells were cultured in Eagle's MEM supplemented with 20% heat‐inactivated FCS, 100 units/mL penicillin, 100 µg/mL streptomycin, 1 mM sodium pyruvate, and 1 mM NEAA. HEK293T cells stably expressing GFP‐LC3B were reported previously [54] and were cultured in DMEM supplemented with 10% (v/v) heat‐inactivated FBS, 6.5 µg/mL gentamicin, and 2 mM l‐glutamine.

### 4.3 Detection of hCoV‐229E and ‐OC43 Nucleocapsid Transcripts

hCoV‐229E and hCoV‐OC43 N transcript levels in the supernatant and cell lysates were determined by RT‐qPCR. Viral RNA in the supernatant and total RNA were extracted using Viral RNA Mini Kit (Qiagen Cat No. 52906) or Quick‐RNA Miniprep Kit (ZYMO research Cat No. R1055) according to the manufacturer's instructions. RT‐qPCR reactions were performed using TaqMan Fast Virus 1‐Step Master Mix (Applied Biosystems Cat No. 4444436) or SuperScript III Platinum One‐Step qRT‐PCR‐Kit (Invitrogen Cat No. 11732088) and a StepOnePlus Real‐Time PCR System. All reactions were run in duplicates. 229E forward: 5′‐TAC TCG AGG TTC CGT CTC GT‐3′; 229E reverse: 5′‐CAA GTG TCA CAC GGT TGC AG‐3′; 229E probe: 5′‐AGC AAA GTT CTG TCT TTG TGG AAA CCA G‐3′; OC43 forward: 5′‐AGC AAC CAG GCT GAT GTC AAT ACC‐3′; OC43 reverse: 5′‐AGC AGA CCT TCC TGA GCC TTC AAT‐3′; OC43 probe: 5′‐TGA CAT TGT CGA TCG GGA CCC AAG TA‐3′. Endogenous control human ACTB (beta actin) primer probes were ordered from Applied Biosystems (Cat No. 4310881E).

### 4.4 Expression Analysis of SERINC Genes

Total RNA was extracted using Quick‐RNA Miniprep Kit (ZYMO research Cat No. R1055), followed by cDNA analysis using PrimeScript RT Reagent Kit with gDNA Eraser (TaKaRa Cat No. RR047B) according to the manufacturer's instructions, 0.5 µg of total RNA was used per reaction. RT‐qPCR was then performed using PowerU SYBR Green Master Mix (Applied Biosystems Cat No. A25918). GAPDH forward: 5′ AAC GGG AAG CTT GTC ATC AAT GGA AA‐3′; GAPDH reverse: 5′‐GCA TCA GCA GAG GGG GCA GAG‐3′; SERINC1 forward: 5′‐AGA TAA TGA AAG GGA TGG TGT C‐3′; SERINC1 reverse: 5′‐ACA GCA CGA TGC CA ATC CAA CT‐3′; SERINC2 forward: 5′‐TGG TGC TGC TCA TCG ACT TT‐3′; SERINC2 reverse: 5′‐TGA AGA AGA AGA GGC CTG CG‐3′; SERINC3 forward: 5′‐AAT TCA GGA ACA CCA GCC TC‐3′; SERINC3 reverse: 5′‐GGT TGG GAT TGC AGG AAC GA‐3′; SERINC4 forward: 5′‐CGT CCT CCA GAG AGA GTA ATC C‐3′; SERINC4 reverse: 5′‐CCA CAG GGG TCC AAA TAC CTC‐3′; SERINC5 forward: 5′‐ATC GAG TTC TGA CGC TCT GC‐3′; SERINC5 reverse: 5′‐GCT CTT CAG TGT CCT CTC CAC‐3′.

### 4.5 SERINC KD Using siRNA Transfection

To KD endogenous SERINC proteins, cells were transfected with siRNA nontargeting control or siRNAs targeting SERINC using lipofectamine RNAiMAX according to manufacturer's instructions when cell density is approximately 70–80%. siGENOME human SERINC1 siRNA (siRNA SERINC1‐01: GGAUUGGCAUCGUGCUGUA; siRNA SERINC1‐02: GGAAUGAAUCGUGGGUUGA; siRNA SERINC1‐03: CGAUGUUCACCGAGCUGUA; siRNA SERINC1‐04: CUGUAUAUC‐GUUUGUGCUU), siGENOME human SERINC2 siRNA (siRNA SERINC2‐01: GGUCAGCCCUAUCCAGUAU; siRNA SERINC2‐02: UCCUGGACUUCGUGCCUUA; siRNA SERINC2‐03: CCUUUGACAACGAGCAGGA; siRNA SERINC2‐04: GCACUGAAG‐CCCUGGUGUU), siGENOME human SERINC3 siRNA (siRNA SERINC3‐01: AAUCAUGG‐GUAAAUCGAAU; siRNA SERINC3‐02: GUGAUGUGCUGGUUGGUUA; siRNA SERINC3‐03: CGUUGUGGCUUCUAUUAUA; siRNA SERINC3‐04: AAAGAGGGAGUGCAGUAUA), siGENOME human SERINC4 siRNA (siRNA SERINC4‐01: CCAGAUAUCUCUCUAGCAA; siRNA SERINC4‐02: CUGCCUGGCCUGAGUAAAA; siRNA SERINC4‐03: CUUCAAGGACAGAAUCACA; siRNA SERINC4‐04: UCUAAGUCCUUUUCACAAA), siGENOME human SERINC5 siRNA (siRNA SERINC5‐01: GGGAUAUUCUGCCGUGUAU; siRNA SERINC5‐02: UGAGAAACCUGAACGGACU; siRNA SERINC5‐03: UGUCAACA‐ACCGUGGCUCA; siRNA SERINC5‐04: CCUCUUAAUCGGAUGUAUC), and siGENOME nontargeting siRNA control pool #2 (CTRL siRNA‐01: UAAGGCUAUGAAGAGAUAC; CTRL siRNA‐01: UAAGGCUAUGAAGAGAUAC; CTRL siRNA‐01: UAAGGCUAUGAAG‐AGAUAC; CTRL siRNA‐04: UGGUUUACAUGUCGACUAA) were ordered from Horizon Discovery.

### 4.6 Tissue Culture Infectious Dose 50

To determine the infectious titer of wild‐type hCoV‐229E and hCoV‐OC43, 10.000 Huh‐7 cells were seeded 1 day before infection in a 96‐well plate. The following day, cells were inoculated with a 10‐fold serial dilution of the respective virus stock. At 3 or 5 days after infection, cytopathic effects were observed by light microscopy and the tissue culture infectious dose 50 (TCID_50_) was calculated with Reed‐Münch method [72].

### 4.7 Preparation of Protein Samples for Western Blot

Cell pellets were lysed in LMNG lysis buffer (150 mM NaCl, 100 mM Tris/HCl pH 8.0, 1% LMNG, 0.2% CHS, 1% DDM, and complete protease inhibitors), mixed with 1×loading buffer and 10% β‐mercaptoethanol and then boiled at 95°C or incubated at room temperature for SERINC proteins for 10 min prior to western blot.

### 4.8 Immunoblotting

After electrophoresis, proteins were transferred to a methanol‐preactivated PVDF membrane using semi‐dry blotting. The membrane and Whatman filter papers were equilibrated in 1× semi‐dry transfer buffer. The gel was rinsed in water, and the transfer sandwich was assembled (filter paper, membrane, gel, filter paper). Transfer was performed at 30 V for 30 min. Following primary antibodies were used in this study: anti‐229E Nucleocapsid (Sinobiological Cat No. 40640‐T62); anti‐OC43 Nucleocapsid (Millipore Cat No. MAB9012); anti‐HA: HA‐Tag (C29F4) rabbit mAb (Cell Signaling Technology Cat No. 3724S); anti‐LAMP1: monoclonal rabbit anti‐LAMP1 antibody, D2D11 (Cell Signaling Technology Cat No. 90919); purified anti‐GAPDH Antibody (Biolegend Cat No. 607902). Secondary antibodies: IRDye 680RD donkey anti‐mouse IgG secondary antibody (LICOR); IRDye 680RD donkey anti‐rabbit IgG secondary antibody (LICOR); IRDye 800CW donkey anti‐mouse IgG secondary antibody (LICOR); IRDye 800CW donkey anti‐rabbit IgG secondary antibody (LICOR); IRDye 680RD goat anti‐rat IgG secondary antibody (LICOR); IRDye 800CW goat anti‐Rat IgG secondary antibody (LICOR). The blots were acquired on Odyssey imager system (LI‐COR Biosciences). Band intensities were quantified using ImageJ.

### 4.9 Incorporation of SERINC Proteins Into Virions

Huh7 cells transiently transfected with plasmids expressing SERINC proteins were infected with hCoV‐229E, ‐OC43, or left untreated. At 72 h postinfection, virus‐containing supernatant was harvested and centrifuged at 1500 rpm for 3 min to remove cell debris, then overlaid on 20% sucrose cushion and centrifuged at 14,000 rpm for 2 h at 4°C. The pellet was dissolved in LMNG lysis buffer supplemented with loading and β‐mercaptoethanol. SERINC proteins, GAPDH and nucleocapsid protein of hCoV‐229E or ‐OC43 were detected by western blot.

### 4.10 Immunofluorescence Staining

For all immunofluorescence staining experiments, cells were seeded on coverslips in 24‐well plates. To determine the subcellular localization of SERINCs in infected cells, cells transfected with plasmids expressing indicated GFP‐SERINC proteins and then infected with HCoV‐229E (MOI 0.01) or ‐OC43 (MOI 0.1) were treated with MG132 (20 µM) for 4 h, NH4Cl (10 mM) for 16 h or left untreated. At 2 days postinfection, cells were washed with PBS and fixed in 4% paraformaldehyde (PFA) solution for 15 min, permeabilized and blocked using PBS containing 0.5% Triton X‐100 and 5% FCS for 40 min at room temperature. Then, cells were washed and incubated with primary antibodies (mouse anti‐229E Nucleocapsid sinobiological 40640‐MM11 at 1:200 or mouse anti‐OC43 Nucleocapsid at 1:100 and rabbit anti‐LAMP1 antibody at 1:200) diluted in PBST at 4°C overnight. Afterward, cells were washed with PBST and incubated with secondary antibodies (donkey anti‐mouse IgG (H + L) Alexa Fluor Plus 568 A10037 and donkey anti‐rabbit IgG (H + L) Alexa Fluor Plus 647 A32795 at 1:400) diluted in PBST at 4°C for 2 h in the dark. Then, cells were washed with PBST and water and mounted onto microscopy slides. Images were acquired with Zeiss LSM 710 confocal laser scanning microscope with ZEN 2010 imaging software (Zeiss). To determine the colocalization of GFP‐SERINC5 and different viral proteins or GFP‐SERINC5 and LAMP1, Huh7 cells were cotransfected with a construct expressing GFP‐SERINC5 and constructs expressing indicated viral proteins. At 2 days posttransfection, cells were harvested and fixed for immunofluorescence staining as described above. Briefly, cells were fixed, permeabilized, blocked, and stained with primary antibodies (mouse Strep Tag II monoclonal antibody at 1:400 and rabbit anti‐LAMP1 antibody at 1:200) at 4°C overnight. Afterward, cells were washed three times with PBST and stained with secondary antibodies (donkey anti‐mouse IgG (H + L) Alexa Fluor Plus 568 A10037 and donkey anti‐rabbit IgG (H + L) Alexa Fluor Plus 647 A32795 at 1:400) for 2 h at 4°C in the dark, washed and mounted onto microscopy slides. To assess the colocalization of SERINC5‐iFLAG and LAMP1 in GFP‐229E‐infected cells, Huh7 were transiently transfected with a construct encoding SERINC5 containing an internal FLAG tag, followed by infection at MOI 0.01. Cells were fixed and permeabilized at 2 days postinfection. Subsequent staining was performed as described above, using mouse monoclonal anti‐FLAG M2 antibody (sigma Cat No. F3165 at 1: 400) and rabbit anti‐LAMP1 antibody (1:200). To determine the infection rate in A549 cells when infected with the supernatant from SERINC KD and infected‐NHLF cells, immunofluorescence staining was performed at 2 days postinfection. Nucleocapsid of OC43 was stained as described above. To assess the subcellular localization of wild‐type and mutant SERINC5, transfected Huh7 cells were fixed, permeabilized, and immune‐stained with a rabbit anti‐HA antibody (Cell signaling Cat No. 3724S at 1:200), followed by a donkey anti‐rabbit IgG (H + L) Alexa Fluor Plus 647 secondary antibody (Invitrogen A32795 at 1:400). Images were acquired using a Leica DMI 8 confocal laser scanning microscope with LAS X Office software.

### 4.11 Colocalization Analysis in Confocal Microscopy

The pictures were analyzed for colocalization of SERINC5‐GFP and LAMP1, SERINC5‐GFP, and indicated viral proteins or SERINC5‐iFLAG and LAMP1 with the Huygens Professional software using the Colocalization Analyzer Advanced module. The colocalization was computed, and the Pearson coefficient was recorded as a measure of colocalization.

### 4.12 Flow Cytometry Analysis of SERINC5 Downregulation by hCoV‐229E and ‐OC43

Huh7 cells transiently transfected with GFP‐SERINC5 were infected with 229E (MOI 0.01) or OC43 (MOI 0.1), followed by NH4Cl treatment (10 mM) for 18 h or MG132 treatment (20 µM) for 4 h. Cells were harvested at 48 h postinfection, washed once with PBS, and stained with eBioscience Fixable Viability Dye eFluor 780 (Thermo Fisher) for 15 min at room temperature in the dark. Afterward, cells were washed twice with PBS and fixed in 4% PFA for 30 min at room temperature. Next, cells were washed twice with PBS and stained with Nucleocapsid antibody rabbit (Sinobiological Cat No. 40640‐T62 for 229E, Cat No. 40643‐T62 for OC43) or rabbit IgG isotype control (Cell Signaling) for 40 min at 4°C. Afterward, cells were washed twice with FACS buffer (1%FCS in PBS), incubated with anti‐rabbit Alexa Fluor 647 conjugated secondary antibody (Thermo Fisher Cat No. A32733) for 30 min at 4°C, washed again, and resuspended in FACS buffer. Flow cytometry was performed on CytoFLEX LX Flow Cytometer (Beckman Coulter Inc.) and analyzed using CytExpert 2.3 software. For western blot analysis, cells were transfected and treated the same as described above and harvested at 48 h posttransfection (hpi).

### 4.13 Flow Cytometry Analysis of SERINC5 Downregulation by Viral Protein of hCoV‐229E

Huh7 cells transiently transfected with a construct expressing GFP‐SERINC5 together with constructs expressing indicated viral proteins were treated with Baf A1 (200 nM) for 12 h, MG132 (20 µM) for 4 h or left untreated before harvest. Cells were harvested for flow cytometry analysis at 24, 28, or 48 h posttransfection. Similarly, cells were washed once with PBS and stained with eBioscience Fixable Viability Dye eFluor 780 for 15 min at room temperature. Afterward, cells were washed and fixed in 4% PFA. After 30 min of fixation at room temperature, cells were then stained with rabbit strep II antibody (Thermofisher Cat No. PA5‐119772) or rabbit IgG isotype control (Abcam Cat No. ab172730) for 1 h at 4°C. Afterward, cells were washed and incubated with anti‐rabbit Alexa Fluor 647 conjugated secondary antibody. After 40 min of incubation at 4°C, cells were washed and resuspended in FACS buffer. Flow cytometry was performed on CytoFLEX LX Flow Cytometer (Beckman Coulter Inc.) and analyzed using CytExpert 2.3 software.

### 4.14 Autophagosome Measurement by Flow Cytometry

HEK293T cells stably expressing GFP‐LC3B cells were transiently reverse transfected with constructs expressing the indicated ORF3a‐like proteins or empty vector in pTwist backbone as described previously [73]. The next day, the supernatant was removed and fresh medium was added. Two days posttransfection the supernatant was discarded and the cells were detached using 0.05%/0.02% trypsin/EDTA. Subsequently, the cells were washed with DBPS and treated with 0.05% saponin in DPBS for 20 min at 4°C for permeabilization as described previously [54, 73]. Afterward, cells were washed twice with DPBS to wash out the nonmembrane bound GFP‐LC3B and fixed with 2% PFA. Autophagosome levels were assessed by detecting the mean fluorescence intensity (MFI) of membrane‐bound GFP‐LC3B via FACS‐Canto II. The MFI value of the empty vector control was used as baseline and subtracted.

### 4.15 Statistics Analysis

Data were collected at least in triplicates for all experiments. For the relative comparison of two samples, statistical differences were assessed by unpaired *t*‐test with Welch's correction. Results were graphed by Graph Pad Prism 9 and are displayed as means ± standard error of the mean (±SEM) with individual values shown. *p* Values ≤ 0.05 were considered statistically significant. A *p* value between 0.05 and 0.01 is annotated with *, *p* values between 0.01 and 0.001 with **, *p* values between 0.001 and 0.0001 with ***, *p* values below 0.0001 with ****.

## Author Contributions

Q.X. and F.K conceived, designed, and supervised experiments. Q.X. performed most experiments with support by S.N., J.L., S.S., S.K., and Q.W, and reviewed, and edited the manuscript. J.M., D.K., and K.M.J.S. provided resources, interpreted data, and edited the manuscript. F.K. conceptualized the study, acquired funding, and wrote the initial draft of the manuscript. All authors have read and approved the final manuscript.

## Funding Information

F.K. is supported by grants from the German Research Foundation (DFG CRC 1279 and CRC 1506) as well as an ERC Advanced Grant (TraitorViruses). K.M.J.S. acknowledges funding by the German Federal Ministry of Education and Research (BMBF; IMMUNOMOD‐01KI2014), the German Research Foundation (DFG; CRC1279, SP 1600/7‐1, SP 1600/9‐1), and the Baden‐Wuerttemberg Stiftung (AutophagyBoost). DFG project ID: 432000323 is acknowledged for the Leica microscope. D.K. was supported by the European Union's Horizon 2020 research and innovation program under Marie Sklodowska‐Curie (grant agreement No. 101062524), German Research Foundation (DFG KM 5/3‐1) and from Else‐Krönung Fresenius Stiftung (2022_EKEA.47).

## Ethics Statement

The authors have nothing to report.

## Conflicts of Interest

The authors declare no conflicts of interest.

## Supporting information




**Supporting File 1**: mco270785‐sup‐0001‐SupMat.pdf

## Data Availability

All data are included in the manuscript or supplements. Source data are provided with this paper. Reagents will be shared by the corresponding author upon reasonable request.
