# Supplementary material for: Human Coronavirus 229E Uses ORF4/4a to Antagonize the Host Restriction Factor SERINC5
Source: MedComm (2020). 2026 May 29;7(6):e70785. doi: 10.1002/mco2.70785 (PMC13240522; doi:10.1002/mco2.70785)
Supplement: Supplementary file 1 — Supporting File 1: mco270785‐sup‐0001‐SupMat.pdf [file MCO2-7-e70785-s001.pdf]

Human coronavirus 229E uses ORF4/4a to antagonize the host restriction factor SERINC5

Short title: hCoV-229E uses ORF4/4a to antagonize SERINC5

Qinya Xie[1], *Sabrina Noettger*[1], *Jan Lawrenz*[1], *Sophie Stopper* [1], *Susanne Klute*[1], *Jan Münch*[1], *Dorota Kmiec*[1], *Qingxing Wang*[1], *Konstantin M.J. Sparrer*[1,2], *Frank Kirchhoff*[1*]

[1] Institute of Molecular Virology
Ulm University Medical Center, 89081 Ulm, Germany
[2] German Center for Neurodegenerative Diseases (DZNE), 89081 Ulm, Germany

Email addresses: 'Qinya Xie' <qinya.xie@uni-ulm.de>; 'Sabrina Noettger' <sabrina.noettger@uni-ulm.de>; 'Jan Lawrenz' <jan.lawrenz@uni-ulm.de>; 'Sophie Stopper' <sophie.stopper@uni-ulm.de>; 'Susanne Klute' <susanne.klute@uni-ulm.de>; Jan Münch <Jan.Muench@uni-ulm.de>; 'Dorota Kmiec' <dorota.kmiec@uni-ulm.de>; 'Qingxing Wang' <wangqingxing16@mails.ucas.ac.cn>; 'Konstantin Sparrer' <konstantin.sparrer@uni-ulm.de>

* Corresponding author:
Frank Kirchhoff, Tel: +49 731 50065150, frank.kirchhoff@uni-ulm.de
ORCID ID: https://orcid.org/0000-0002-7052-2360

Field codes: Virology, Innate immunity

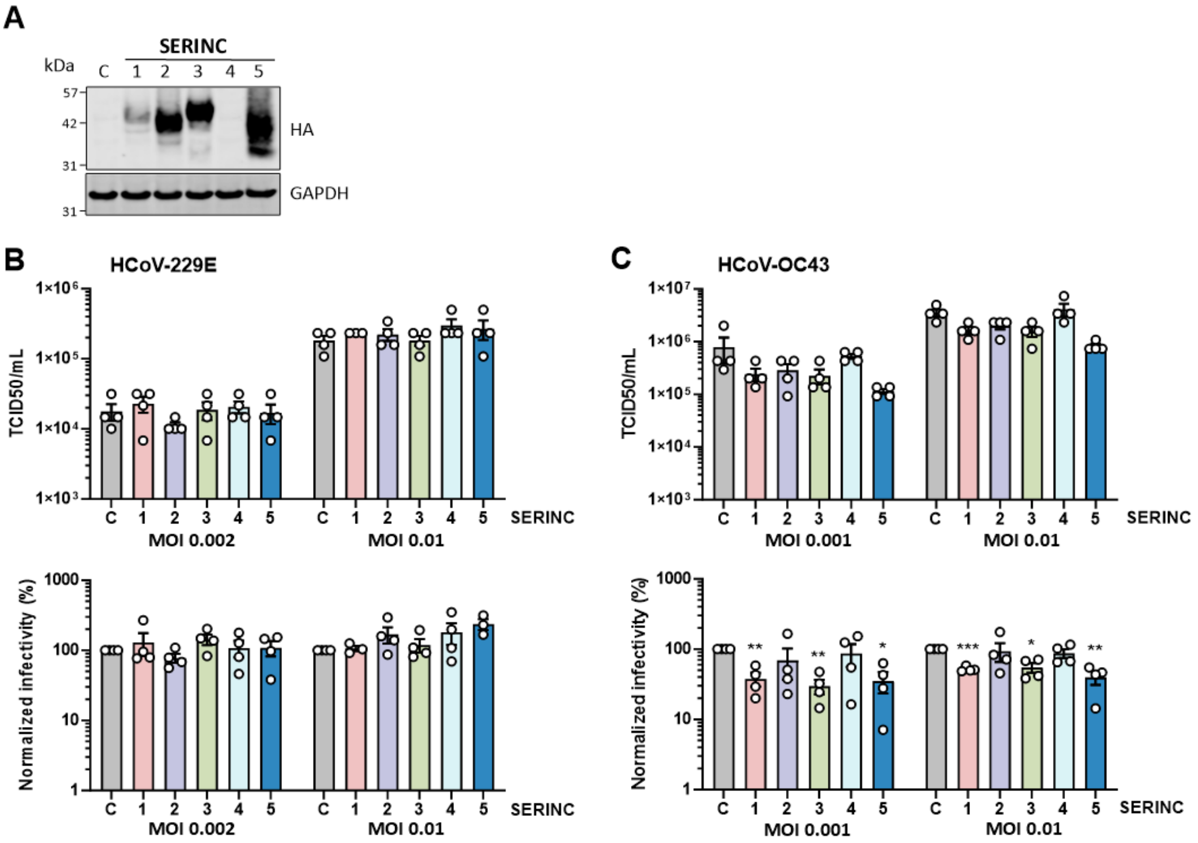

Figure S1. SERINCs reduce the infectivity of hCoV-OC43 but not hCoV-229E. (A) Huh7 cells were transfected with plasmids expressing HA-tagged SERINC1-5. Whole cell lysates were harvested at 48 hours post-transfection for western blot. **(B, C)** Absolute $TCID_{50}$ values and infectivity normalized to viral RNA copy numbers shown in Figures 1C and 1D. Normalized infectivity was calculated as $TCID_{50}$/vRNA copy number, with the vector control set to 100%. Data represent mean values ± SEM from three or four independent experiments. Statistical significance compared to the mock control was assessed using unpaired t-test with Welch`s correction. $^{*}p \leq 0.05$, $^{**}p \leq 0.01$, $^{***}p \leq 0.001$.

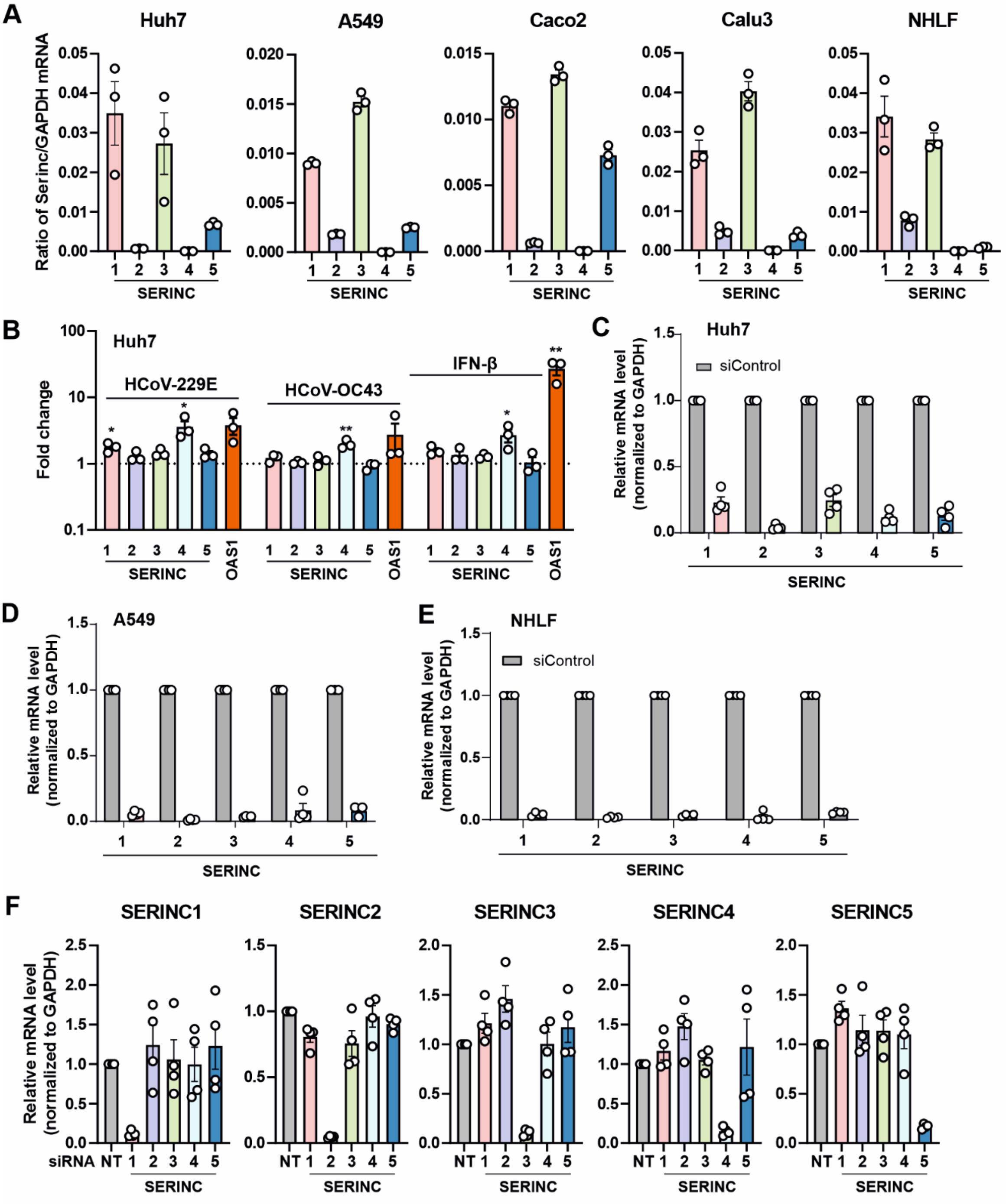

Figure S2. SERINC mRNA expression levels and knock-down efficiencies. (A) Total RNA was extracted from Huh7, A549, Caco2, Calu3 and NHLF cells, followed by reverse transcription and SYBR Green qPCR to quantify SERINC1, 2, 3, 4 and 5 mRNA levels relative to GAPDH mRNA levels. **(B)** Huh7 cells were treated with IFN-β (100 IU/ml), infected with hCoV-229E (MOI 0.001) or HCoV-OC43 (MOI 0.01), or left untreated. Total RNA was collected at 48 hours post-treatment and analyzed by SYBR Green qPCR for SERINC1–5 and OAS1 (interferon-stimulated gene control). Fold changes were calculated relative to untreated cells. **(C-E)** Knock-down efficiency of SERINCs in Huh7 **(C)**, A549 **(D)** and NHLF **(E)** was determined by reverse transcription and qPCR. **(F)** siRNA specificity for individual SERINCs was validated by qPCR. Data represent mean values ± SEM from three independent experiments. Statistical significance of differences compared to the mock control was determined using unpaired t-test with Welch`s correction. $*p \leq 0.05$, $**p \leq 0.01$.

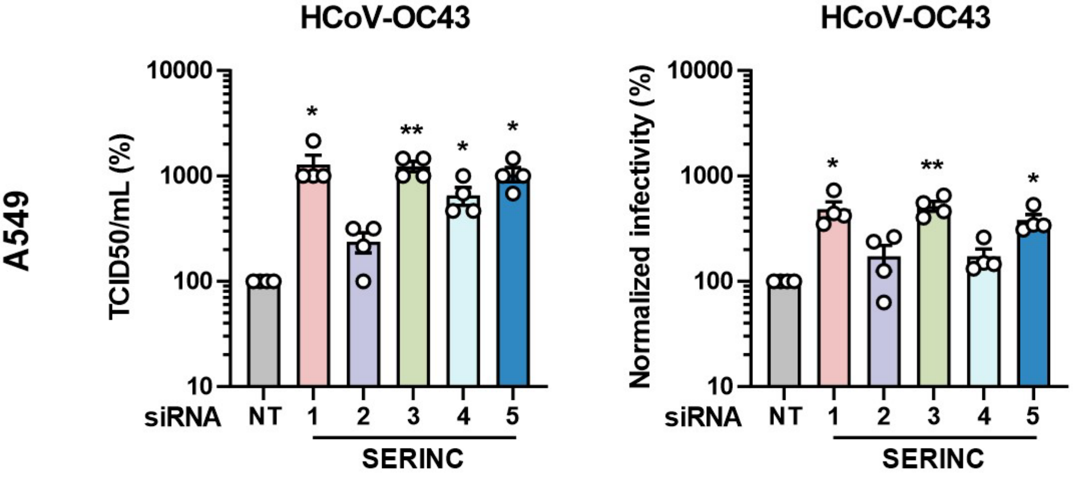

Figure S3. Endogenous SERINC proteins restrict the infectivity of HCoV-OC43 in A549. A549 cells transiently transfected with non-targeting or SERINC targeting siRNA were infected with hCoV-OC43 at MOI 0.01. Supernatants were collected for qRT-PCR and $TCID_{50}$ at 72 hours post-infection. Normalized infectivity was calculated as $TCID_{50}$/vRNA copy number, with the vector control set to 100%. Data represent mean values ± SEM from four independent experiments. Statistical comparisons to mock control were performed using unpaired t-test with Welch's correction. *$p \leq 0.05$; **$p \leq 0.01$.

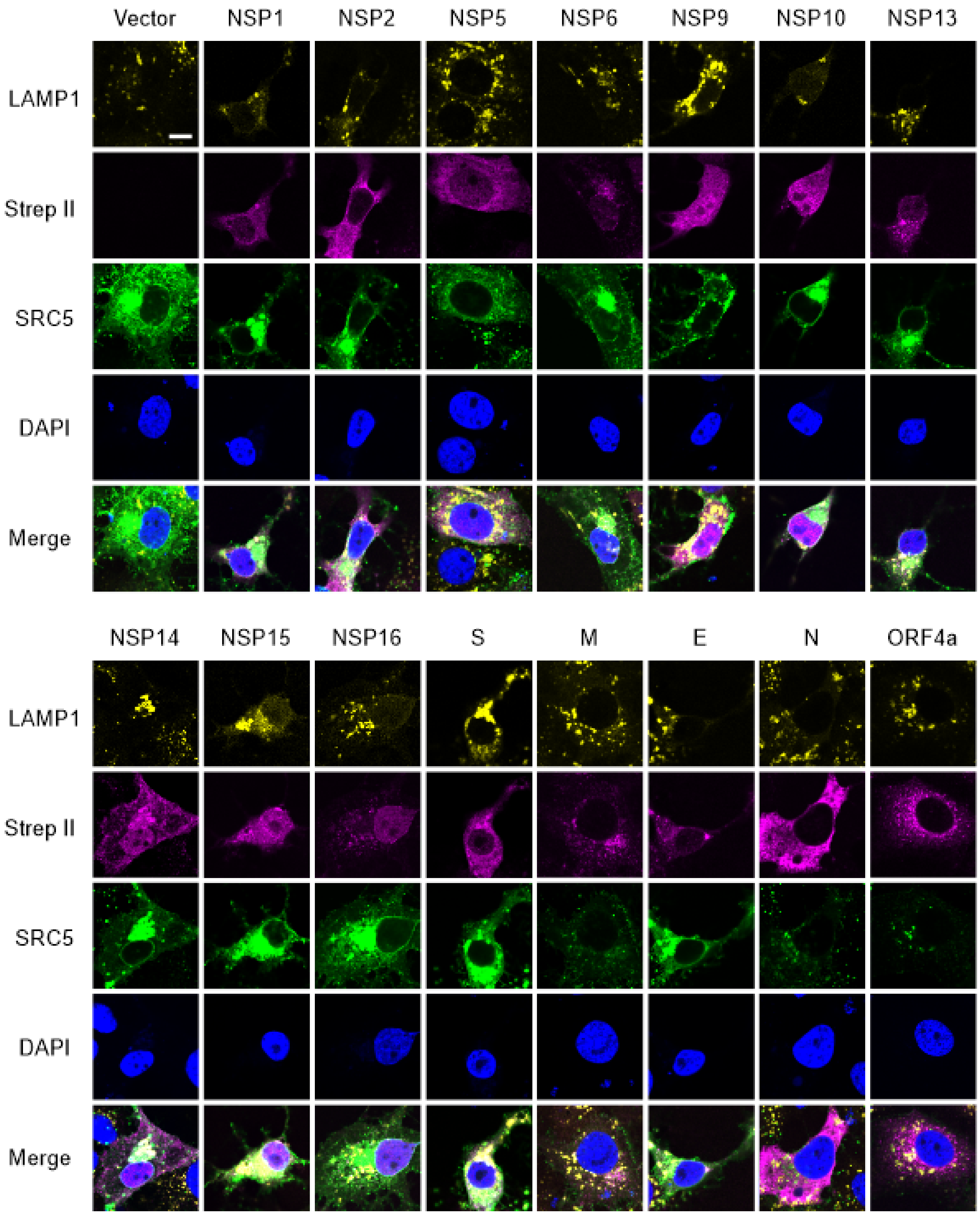

Figure S4. Subcellular localization of GFP-SERINC5 in the presence of hCoV-229E proteins. Huh7 cells were co-transfected with constructs expressing GFP-SERINC5 and the indicated hCoV-229E viral proteins. At 2 days post-transfection, cells were fixed for immunofluorescence staining. Strep II (purple) marks expression of viral proteins; LAMP1 (yellow) labels lysosomes; nuclei were stained with DAPI (blue). Scale bar: 20 µm. Co-localization of GFP-SERINC5 with LAMP1 or with Strep II–tagged viral proteins was analyzed in 10–22 cells per condition.

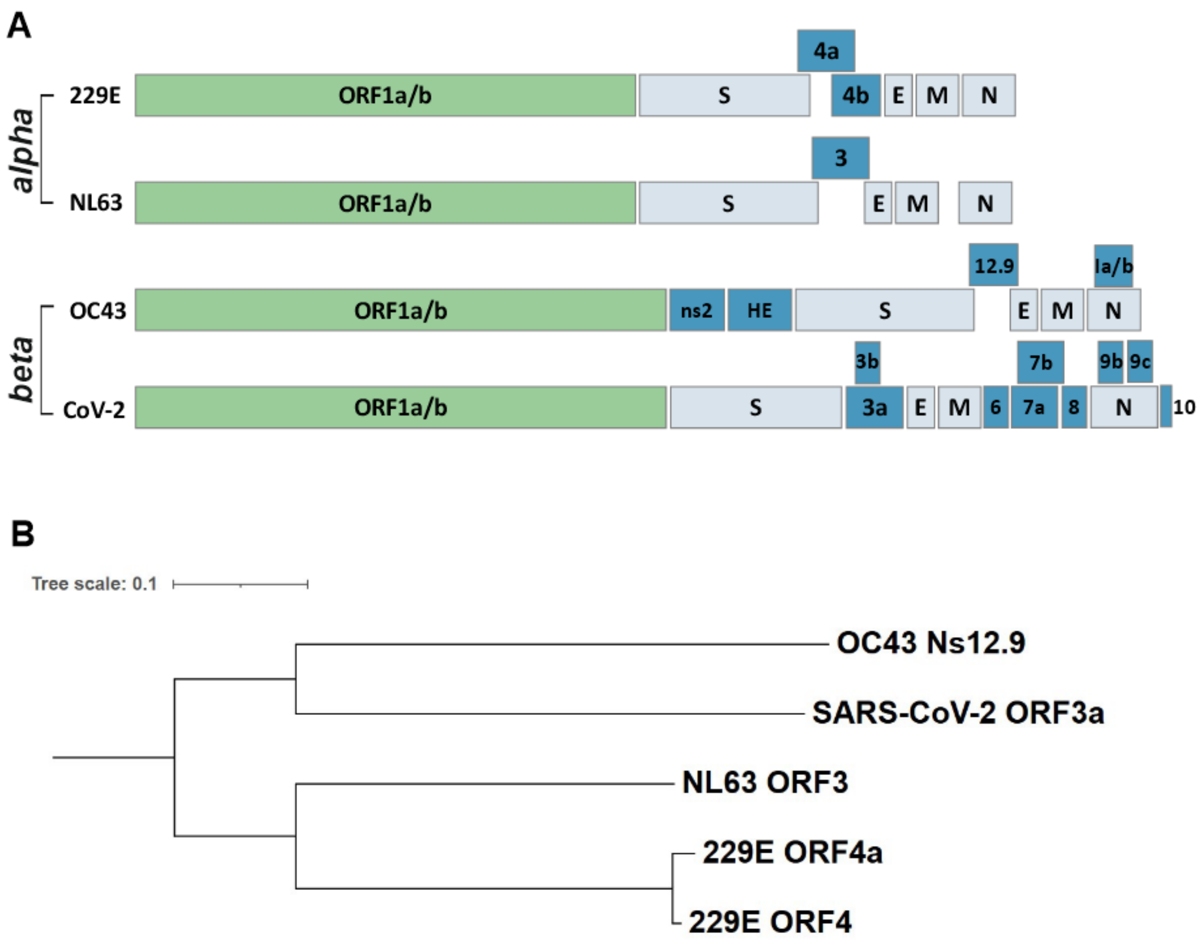

Figure S5. Genomic organization and evolutionary comparison of selected coronavirus accessory proteins. (A) Genomic structures of hCoV-229E, hCoV-NL63, hCoV-OC43, and SARS-CoV-2. **(B)** Nucleotide sequences of coronavirus genes were obtained from the NCBI database (GenBank: NC_002645.1; PQ187611.1; NC_005831.2; NC_045512.2; NC_006213.1). Phylogenetic trees showing distance-based relationship inference based on nucleotide sequences were generated using the NGPhylogeny.fr FastME tool (https://ngphylogeny.fr/) and were visualized using iTOL (https://itol.embl.de/).

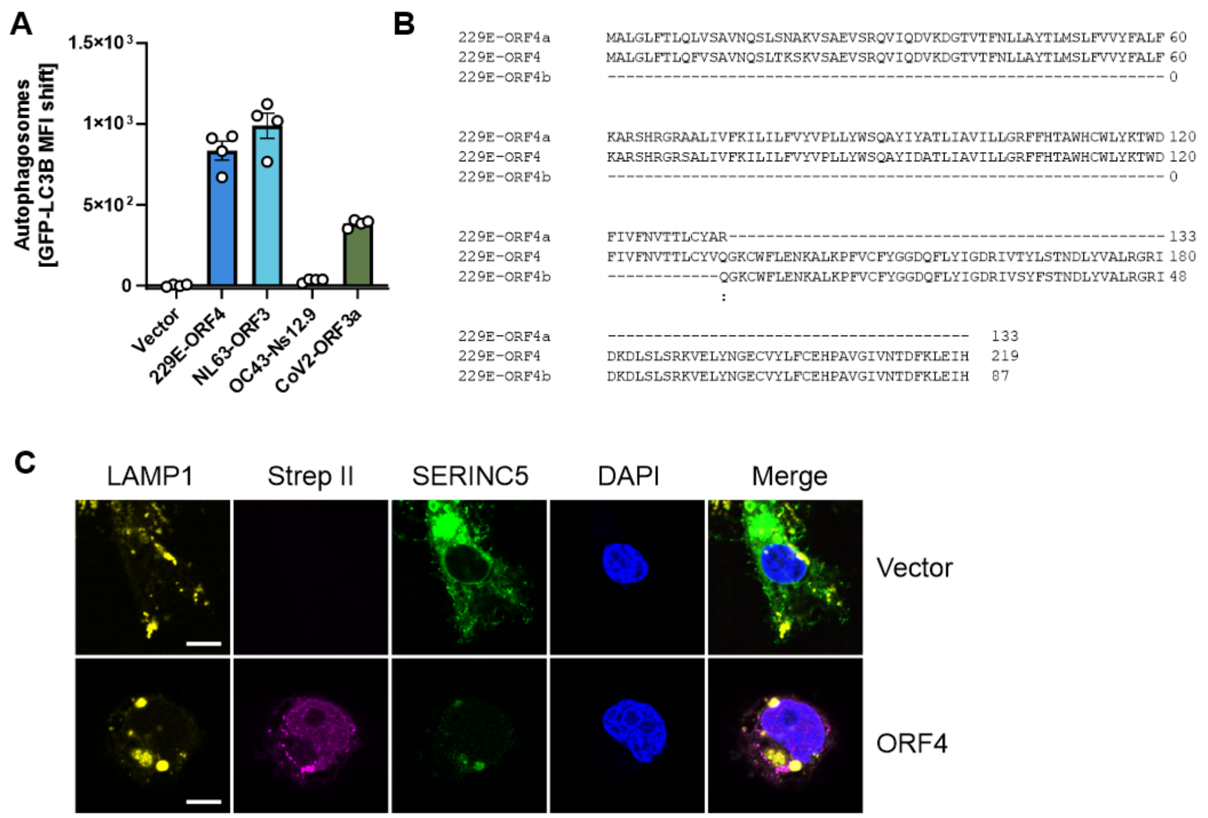

Figure S6. Sequence and function of 229E-ORF4. **(A)** Quantification of autophagosome levels in autophagy reporter cells (HEK293T GFP-LC3B) transfected with empty vector or constructs expressing the indicated ORF3a-like proteins (2 days post transfection). GFP-LC3B mean fluorescence intensity (MFI) was measured by flow cytometry to assess autophagy modulation. Bars represent mean values ± SEM from four biological replicates. **(B)** Amino acid sequences of HCoV-229E-ORF4a, -ORF4b and -ORF4 were aligned using Clustal Omega. (**C**) Huh7 cells were co-transfected with constructs expressing GFP-SERINC5 and an empty control or 229E ORF4-expressing vector. At 2 days post-transfection, cells were fixed for IF staining. Strep II tag (purple) marks ORF4 expression; LAMP1 (yellow) labels lysosomes; nuclei were stained with DAPI (blue). Scale bar: 20 µm.

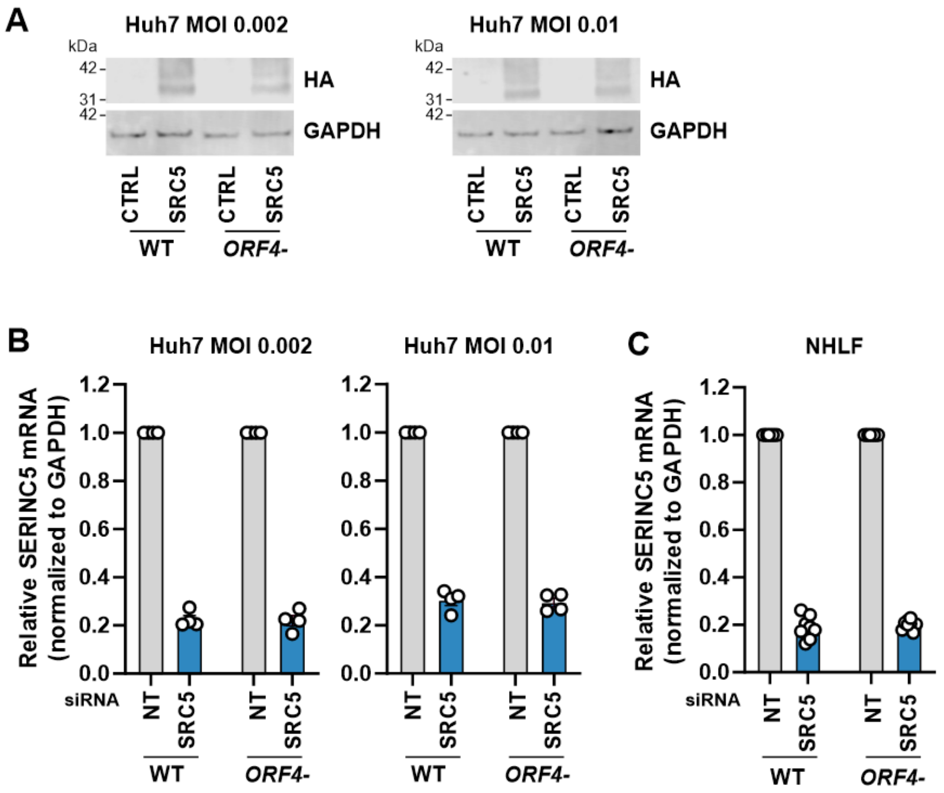

Figure S7. Confirmation of SERINC5 overexpression and knock-down. (A) SERINC5 overexpression in Huh7 cells was detected by western blot analysis. **(B, C)** Knock-down efficiency of SERINC5 in Huh7 cells **(B)** and NHLF **(C)** was determined by reverse transcription followed by qPCR.
